# Tumor analysis of MMR genes in Lynch‐like syndrome: Challenges associated with results interpretation

**DOI:** 10.1002/cam4.7041

**Published:** 2024-04-01

**Authors:** Paula Rofes, Núria Dueñas, Jesús del Valle, Matilde Navarro, Judith Balmaña, Teresa Ramón y Cajal, Noemí Tuset, Carmen Castillo, Sara González, Joan Brunet, Gabriel Capellá, Conxi Lázaro, Marta Pineda

**Affiliations:** ^1^ Hereditary Cancer Program Catalan Institute of Oncology, Molecular Mechanisms and Experimental Therapy in Oncology Program, Institut d'Investigació Biomèdica de Bellvitge – IDIBELL L'Hospitalet de Llobregat Spain; ^2^ Centro de Investigación Biomédica en Red de Cáncer (CIBERONC) Madrid Spain; ^3^ Hereditary Cancer Genetics Group, Vall d'Hebron Institute of Oncology (VHIO) Vall d'Hebron Hospital Barcelona Spain; ^4^ Medical Oncology Department Hospital de la Santa Creu i Sant Pau Barcelona Spain; ^5^ Medical Oncology Department Arnau de Vilanova University Hospital Lleida Spain; ^6^ Hereditary Cancer Program Catalan Institute of Oncology – IDIBGi Girona Spain

**Keywords:** clinical management, Lynch syndrome, Lynch‐like syndrome, mismatch repair genes, mismatch repair‐deficiency, tumor testing

## Abstract

**Background:**

Up to 70% of suspected Lynch syndrome patients harboring MMR deficient tumors lack identifiable germline pathogenic variants in MMR genes, being referred to as Lynch‐like syndrome (LLS). Previous studies have reported biallelic somatic MMR inactivation in a variable range of LLS‐associated tumors. Moreover, translating tumor testing results into patient management remains controversial. Our aim is to assess the challenges associated with the implementation of tumoral MMR gene testing in routine workflows.

**Methods:**

Here, we present the clinical characterization of 229 LLS patients. MMR gene testing was performed in 39 available tumors, and results were analyzed using two variant allele frequency (VAF) thresholds (≥5% and ≥10%).

**Results and Discussion:**

More biallelic somatic events were identified at VAF ≥ 5% than ≥10% (35.9% vs. 25.6%), although the rate of nonconcordant results regarding immunohistochemical pattern increased (30.8% vs. 20.5%). Interpretation difficulties question the current utility of the identification of MMR somatic hits in the diagnostic algorithm of suspected LS cases.

## Graphical abstract

Biallelic somatic MMR inactivation has been reported in a variable range of LLS‐associated tumors. More biallelic somatic events are identified with a lower VAF threshold, although the rate of nonconcordant results regarding immunohistochemical pattern increase. Interpretation difficulties question the current utility of the identification of MMR somatic hits in the diagnostic algorithm of suspected LS cases.

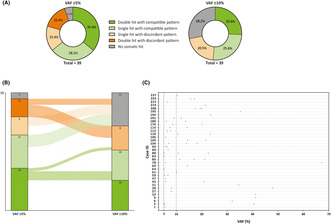

## 1 INTRODUCTION

Lynch syndrome (LS) accounts for ~3% of colorectal cancer (CRC) cases and also predisposes to endometrium, ovary, and other neoplasms.^1^ LS is caused by germline inactivation of one of the mismatch repair (MMR) genes (*MLH1*, *MSH2*, *MSH6*, or *PMS2*) or *EPCAM* gene deletions, and its molecular diagnosis mainly relies on MMR‐deficiency (MMRd) screening in tumors (microsatellite instability [MSI] assessment and/or MMR immunohistochemical [IHC] staining) along with germline MMR testing.^2^ Up to 70% of patients harboring MMRd tumors not linked to somatic methylation of MMR genes lack identifiable germline pathogenic variants (PV).^3^, ^4^ These cases are referred to as Lynch‐like syndrome (LLS). Recent studies have reported somatic biallelic inactivation of MMR genes at frequencies ranging from 15% to 95% of LLS tumors (reviewed in Table S1), likely representing sporadic events. Additionally, MMRd can result from germline biallelic inactivation of *MUTYH* or *MSH3* or germline PVs in the exonuclease domains of *POLD1* and *POLE* genes.^5^, ^6^, ^7^

Clinical management of LLS is challenging, since they represent a heterogeneous group of hereditary and sporadic conditions. LLS relatives have a lower risk of CRC compared with those from LS individuals, but higher than sporadic cases.^3^ In this work, we conducted a clinical and molecular characterization of our LLS cohort and performed tumor testing in patients with available tumors. Our aim was to assess the viability of implementing tumor analyses in routine genetic testing workflows to improve the diagnosis and management of LLS patients.

## 2 PATIENTS AND METHODS

### 2.1 Patients

The complete clinical cohort comprises 229 LLS cases (52.4% males and 47.6% females): 105 previously reported^8^ and 124 newly identified patients with MMRd and/or MSI in tumors, but no germline MMR PVs nor somatic hypermethylation of *MLH1* promoter (Figure S1). Clinical and tumor data are displayed in Table 1 and Table S2.

**TABLE 1:** Summary of demographics, tumoral, and molecular data from a series of 229 Lynch‐like individuals.

	All	Male	Female
	Total *n* (%)	Total *n* (%)	Total *n* (%)
Demographics			
Complete cohort	229 (100%)	120 (52.4%)	109 (47.6%)
Mean current age (range)	64 (21–95)	64 (21–95)	64 (26–94)
Deceased	49 (21.4%)	27 (22.5%)	22 (9.6%)
Family history			
*Yes*	79 (34.9%)	42 (35.0%)	37 (33.9%)
Amsterdam II	13 (6.1%)	5 (4.2%)	8 (7.3%)
*No*	150 (65.5%)	78 (65.0%)	72 (66.1%)
Tumoral data			
*Mean age at first cancer*	54.1 (16–85)	54.8 (16–85)	53.3 (24–81)
*Mean age at LS‐related tumor*	55.2 (16–85)	56.7 (16–85)	55.1 (24–81)
LS‐related tumor <50 years	82 (35.8%)	42 (35.0%)	40 (36.7%)
With family history	26 (31.7%)	13 (31.0%)	13 (32.5%)
Without family history	56 (68.3%)	29 (69.0%)	27 (67.5%)
LS‐related tumor >50 years	147 (64.2%)	78 (65.0%)	69 (63.3%)
With family history	53 (36.1%)	29 (37.2%)	24 (34.8%)
Without family history	94 (63.9%)	49 (62.8%)	45 (65.2%)
*Individuals with CRC*	207 (90.8%)	116 (96.7%)	91 (83.5%)
Location of CRC			
Proximal colon	122 (58.9%)	57 (49.1%)	65 (71.4%)
Distal colon	60 (29.0%)	42 (36.2%)	18 (19.8%)
Multiple locations	22 (10.6%)	15 (12.9%)	7 (7.7%)
Unknown	3 (1.4%)	2 (1.7%)	1 (1.1%)
*Individuals with EC*	21 (9.2%)	0 (0%)	21 (19.3%)
*Individuals with other LS‐related tumors*	13 (5.7%)	7 (5.8%)	6 (5.5%)
*Individuals with other LS‐nonrelated tumors*	31 (13.5%)	19 (15.7%)	12 (11.0%)
*Individuals with multiple tumors*	58 (25.3%)	33 (27.5%)	25 (22.9%)
*Stage at diagnosis of MMRd/MSI tumors*			
I	36 (15.7%)	13 (10.8%)	23 (21.1%)
II	110 (48.0%)	68 (56.7%)	42 (38.5%)
III	53 (23.1%)	25 (20.8%)	28 (25.7%)
IV	10 (4.4%)	6 (5.0%)	4 (3.7%)
Unknown	20 (8.7%)	8 (6.7%)	12 (11.0%)
Molecular data			
*MMRd*	205 (89.5%)	104 (86.7%)	101 (92.7%)
Loss MLH1‐PMS2	122 (59.5%)	60 (57.7%)	62 (61.4%)
Loss PMS2	7 (3.4%)	5 (4.7%)	2 (2.0%)
Loss MSH2‐MSH6	52 (25.4%)	27 (26.0%)	25 (24.8%)
Loss MSH6	24 (11.7%)	12 (11.5%)	12 (11.9%)
*MSI*	128 (55.9%)	66 (28.8%)	62 (56.9%)

Abbreviations: CRC: colorectal cancer; EC: endometrial cancer; LS, Lynch syndrome; LS‐related tumor: in our cohort includes colorectal cancer, endometrial cancer, ovarian cancer, gastric cancer, small intestine cancer and urothelial cancer; MMRd: mismatch repair deficient; MSI: microsatellite instability.

### 2.2 Tumor testing

In 41/229 LLS patients, tumor samples with >70% tumor cellularity were available for molecular testing and fulfilled the following inclusion criteria: (1) MSI and/or MMRd tumors; (2) no germline (likely) PVs in *MLH1*, *MSH2*, *MSH6*, *PMS2*, or *EPCAM* identified, which were sequenced based on their IHC MMR expression pattern; and (3) no somatic *MLH1* promoter hypermethylation (Table S3). DNA extracted from colorectal or endometrial carcinomas was analyzed using our I2HCP next‐generation sequencing (NGS) panel adapted for somatic testing.^9^ All variants identified in coding and adjacent intronic regions (i.e., ±20 bp) at variant allele frequencies (VAFs) ≥5% and covered at ≥30X were retrieved from the following genes: *MLH1* (NM_000249.3), *MLH3* (NM_001040108.1), *MSH2* (NM_000251.1), *MSH3* (NM_002439.3), *MSH6* (NM_000179.2), *MUTYH* (NM_001128425.1), *PMS2* (NM_000535.5), *POLD1* (NM_001256849.1), and *POLE* (NM_006231.2). Variant classification was performed using vaRHC, an in‐house developed tool for the semi‐automation of variant interpretation according to ACMG/AMP ClinGen's gene‐specific guidelines.^10^ Whenever possible, loss of heterozygosity (LOH) was assessed by comparing the allelic fraction of germline heterozygous SNPs in paired tumor DNA. LOH status was considered positive if at least three constitutionally heterozygous SNPs displayed an allelic imbalance in tumor tissue.^11^ Variants identified in tumors at VAFs ≥20% were retested in blood‐derived DNA by Sanger sequencing to discard constitutional variants previously missed as recommended.^12^

## 3 RESULTS AND DISCUSSION

Our clinical series comprises 229 LLS individuals. 34.9% had a family history of LS‐related tumors, and there was no association between age at LS‐related tumors and family history. The mean age at first cancer diagnosis was 53.9 years (range 16–85). The majority were diagnosed with CRC (90.8%), predominantly in the proximal colon (58.9%), 19.3% of women were also diagnosed with endometrial cancer, and 5.7% were diagnosed with other tumors related to LS (ovarian, gastric, small intestine, and urothelial cancers). The IHC pattern of MMRd was coincident with other published results, with a significant proportion demonstrating a lack of MLH1‐PMS2 expression (59.5%) and MSH2‐MSH6 expression (25.4%). Table 1 summarizes demographics, tumor, and molecular data; Table S2 displays the complete information from the series.

**FIGURE 1:**
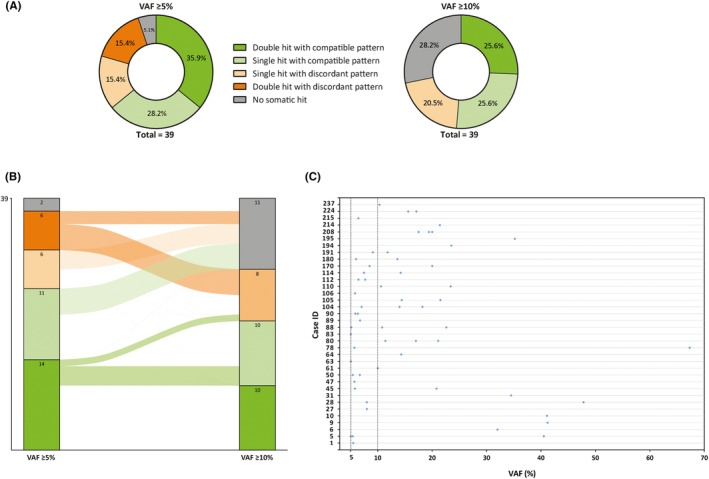
Summary of results from tumor analyses in the somatic testing cohort. (A) Pie charts displaying the interpretation of somatic testing results according to immunohistochemical staining of tumors of 39 LLS individuals using two different VAF thresholds: ≥5% and ≥10%. (B) Sankey diagram showing the divergences in the interpretation of somatic testing results in 39 LLS individuals using two different VAF thresholds. Scaled arcs are used to display flows in the interpretation from VAF ≥5% scenario to VAF ≥10% scenario. (C) Scatter plot displaying somatic MMR hits identified in 39 LLS individuals. Cases are represented in the Y axis, VAFs are indicated in the X axis, and MMR hits concordant with immunohistochemical staining of tumors are plotted as blue dots. Two vertical discontinuous lines delimitate MMR hits identified at VAFs ranging from 5% to 10%, in order to highlight somatic MMR hits lost when raising the VAF threshold from ≥5% to ≥10%. LLS, Lynch‐like syndrome; VAF, variant allele frequency.

Out of 41 LLS tumors, two samples (4.9%) were discarded due to failed DNA amplification. By using a VAF threshold ≥5%, in 14/39 cases (35.9%), double somatic hits consistent with the IHC patterns were identified: in 10/14 (71.4%), two somatic MMR PVs were detected, while 4/14 (28.6%) showed one somatic MMR PV along with LOH of the same MMR gene. In addition, some patients harbored more than two somatic hits affecting the same MMR gene (Cases 5, 104, 208). Furthermore, in 11/39 (28.2%) samples, one somatic hit consistent with the tumor MMR IHC pattern was detected. LOH could not be evaluated in nine of them due to the lack of informative SNPs, since pre‐NGS genetic testing algorithms were directed to only one or two MMR genes. In 12/39 (30.8%) cases, the somatic hits identified disagreed with the IHC pattern. Additionally, four patients with one compatible somatic hit harbored additional somatic PVs in other MMR genes, nonconcordant with the tumor MMR IHC pattern, making interpretation challenging (Cases 45, 61, 78, 194) (Figure 1A; Table S3).

To address interpretation ambiguities and due to the lack of orthogonal validation, VAF detection threshold was raised to ≥10%^13^ to minimize potential false positive calls and passenger mutations that are the effect of MSI rather than cause.^14^ With this adjustment, 10/39 patients (25.6%) harbored compatible double somatic hits according to their IHC MMR status (compared with 35.9% in the previous scenario). The rate of nonconcordant results also decreased significantly (30.8% to 20.5%). However, the number of patients with no somatic hits identified increased (5.1% to 28.2%) (Figure 1A–C).

No PVs were identified in other genes that may contribute to MMRd, such as *POLE*, *POLD1*, *MSH3*, or *MUTYH*.

As summarized in Table S1, there is a wide heterogeneity in the percentage of biallelic MMR inactivation reported, which can be partly attributable to differences in the experimental designs and data analysis, making it difficult to draw unbiased comparisons. Besides, the limitations derived from the interpretation of somatic analyses have not yet been clearly depicted. Considering hypermutability in MMRd tumors, ambiguous interpretations may arise, which evidence the necessity of standardized protocols. Our study shows that a single tumor may present several hits involving different MMR genes. Furthermore, in 30.8% of cases (VAF ≥5%) or 20.5% of cases (VAF ≥10%), the predominant MMR hit disagreed with the IHC pattern. These cases were considered inconclusive.

**FIGURE 2:**
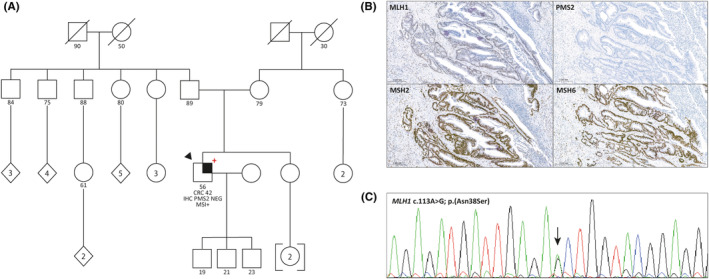
Details from a Lynch syndrome patient identified through tumor testing (Case 198). (A) Pedigree from Case 198. A black arrowhead is used to indicate the proband, and a red plus symbol to display carrier status of *MLH1* c.113A > G pathogenic variant. A quarter of an individual's symbol shadowed represents individuals diagnosed with cancer. The current age of each individual is displayed below their symbol, followed by their cancer type and age of onset. (B) Immunohistochemistry screening of mismatch repair genes *MLH1*, *MSH2*, *MSH6* and *PMS2*. Results indicate loss of PMS2 expression. (C) Blood testing by Sanger sequencing in patient 198 confirms the presence of *MLH1* c.113A > G as a constitutional variant previously missed. CRC, colorectal cancer; IHC, immunohistochemistry; MSI+, microsatellite instability positive (high); NEG, negative IHC pattern (loss of expression).

A remarkable finding of this study is illustrated in Case 198 (Figure 2A). Routine tumor screening identified PMS2 loss of expression (Figure 2B), prompting only *PMS2* germline mutational analysis. However, *MLH1* c.113A > G; p.(Asn38Ser) PV was detected at VAF = 59.1% in tumor DNA and was subsequently confirmed in blood DNA (Figure 2C), resulting in the reclassification of patient 198 as LS. This finding highlights one of the drawbacks of former genetic testing strategies, which targeted the candidate MMR gene according to the IHC pattern. More recently, the advent of NGS technologies has resulted in the implementation of multigene panels in most diagnostic workflows, allowing simultaneous analysis of all MMR genes in patients with suspected LS. Nevertheless, the analysis of *PMS2* using NGS‐based approaches still poses challenges in the diagnostic setting due to the presence of highly homologous pseudogenes.^15^

In tumor analyses, the resulting yield highly depends on the quality of FFPE DNA. Our approach has detected somatic hits with a robust mean coverage of 193X, ensuring the reliability of our findings. However, the highly variable coverage across different genomic regions precluded the analysis of copy‐number variants. The use of gene panels specifically designed for FFPE samples may allow the assessment of other mechanisms of somatic inactivation unexplored in our series.

Paired somatic‐germline testing has been repeatedly suggested as a useful approach to increase the diagnostic yield by identifying germline PV causing LS as well as biallelic MMR somatic mutations in LLS tumors. Besides, paired analyses may ease the interpretation of MMR variants, particularly of those detected at low VAF. This strategy also holds potential for detecting somatic mosaicism, although mosaic mutations in MMR genes appear to be rare.^16^, ^17^

As shown in our work, the interpretation of tumor analyses in individuals with LLS is sometimes controversial. By using our approach, double somatic MMR inactivation could only explain up to one‐third of MMRd tumors of our series. Moreover, no consensus has been reached as to whether LLS patients with no biallelic MMR variants identified should be treated as having LS^18^, ^19^ or treated based on personal and family history.^2^, ^3^, ^20^ In the era of multigene testing, a germline cause can be ruled out with high probability when noninformative results are obtained. This fact, added to the difficulties in the interpretation of somatic analyses and the lack of consensus of its impact in the clinical management of LLS patients, poses an interrogation in the current utility of MMR somatic hit identification in the routine diagnostic algorithm of suspected LS cases. In the context of routine diagnosis, we recommend restricting the analysis of somatic variants in MMR genes in LLS tumors only after verifying that the approach used demonstrates a high rate of informative results.

In the era of multigene testing, a germline cause is ruled out with high probability when noninformative results are obtained. This fact, added to the difficulties in the interpretation of somatic analyses and the lack of consensus of its impact in the clinical management of LLS patients, poses an interrogation in the current utility of MMR somatic hit identification in the routine diagnostic algorithm of suspected LS cases.

## AUTHOR CONTRIBUTIONS

**Paula Rofes:** Conceptualization (lead); data curation (equal); formal analysis (lead); investigation (lead); methodology (lead); software (lead); writing – original draft (lead); writing – review and editing (lead). **Núria Dueñas:** Conceptualization (lead); data curation (lead); formal analysis (lead); investigation (lead); methodology (lead); software (equal); writing – original draft (lead); writing – review and editing (lead). **Jesús del Valle:** Formal analysis (supporting); writing – original draft (supporting); writing – review and editing (equal). **Matilde Navarro:** Conceptualization (supporting); data curation (supporting); writing – review and editing (equal). **Judith Balmaña:** Data curation (supporting); writing – review and editing (equal). **Teresa Ramón y Cajal:** Data curation (supporting); writing – review and editing (equal). **Noemí Tuset:** Data curation (supporting); writing – review and editing (equal). **Carmen Castillo:** Writing – review and editing (equal). **Sara González:** Formal analysis (supporting); writing – review and editing (supporting). **Joan Brunet:** Data curation (supporting); writing – review and editing (equal). **Gabriel Capellá:** Writing – review and editing (equal). **Conxi Lázaro:** Conceptualization (equal); formal analysis (equal); investigation (equal); methodology (equal); supervision (equal); validation (equal); writing – original draft (equal); writing – review and editing (equal). **Marta Pineda:** Conceptualization (equal); investigation (equal); methodology (equal); supervision (equal); validation (equal); writing – original draft (equal); writing – review and editing (equal).

## FUNDING INFORMATION

This study was supported by Carlos III National Health Institute and Ministerio de Ciencia e Innovación and funded by FEDER funds—a way to build Europe [PI19/00553; PID2019‐111254RB‐I00]; CIBERONC [CB16/12/00234]; and the Government of Catalonia (Secretariat for Universities and Research of the Department of Business and Knowledge, grant 2021SGR01112).

## CONFLICT OF INTEREST STATEMENT

Authors declare no conflict of interest.

## ETHICAL APPROVAL

Informed written consent for both diagnostic and research purposes was obtained from all patients. The study protocol was approved by the ethics committee of Bellvitge Biomedical Research Institute (IDIBELL; PR278/19).

## Supporting information


Figure S1

Table S1

Table S2

Table S3

## Data Availability

All the data generated in this study can be found within the published article and its supporting information (Tables [Link], [Link]) and (Figure S1).
