# Supplementary figures and images for: Tumor analysis of MMR genes in Lynch‐like syndrome: Challenges associated with results interpretation
Source: Cancer Med. 2024 Apr 1;13(7):e7041. doi: 10.1002/cam4.7041 (PMC10983805; doi:10.1002/cam4.7041)

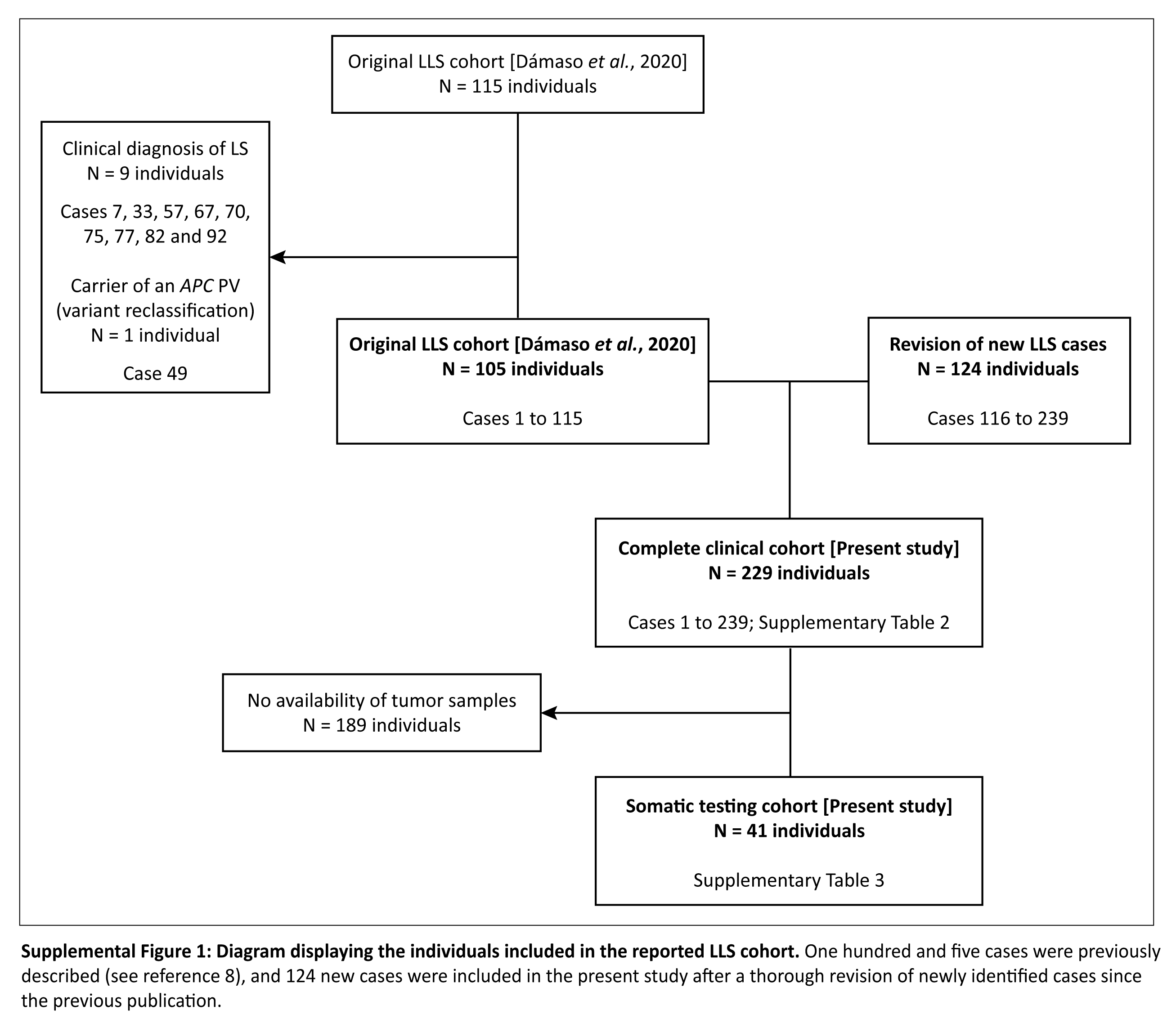

Supplement: Supplementary file 1 — Figure S1 [file CAM4-13-e7041-s001.tif]
